# Efficacy of frog skin lipids in wound healing

**DOI:** 10.1186/1476-511X-9-74

**Published:** 2010-07-19

**Authors:** Venkat Raghavan K, Mary Babu, Rama Rajaram, Korrapati Purna Sai

**Affiliations:** 1 Department of Biomaterials, Central Leather Research Institute, Chennai, India

## Abstract

**Background:**

Frog skin has been sequentially and scientifically evaluated by our group for its wound healing efficiency. Owing to the complex structure of skin, attempts were being made to analyse the role of individual constituents in different phases of healing. Our earlier papers have shown the significance of frog skin not only in wound healing but also enhancing the proliferating activity of the epidermal and dermal cells which are instrumental for normal healing process. We also have identified for the first time novel antimicrobial peptides from the skin of *Rana tigerina *and thereby reduce the complications involved in the sepsis.

**Purpose of the study and Results:**

The current study envisages the role of frog skin lipids in the inflammatory phase of wound healing. The lipid moiety of the frog skin dominated by phospholipids exhibited a dose dependent acceleration of healing irrespective of the mode of application. The efficiency of the extract is attributed partially to the anti-inflammatory activity as observed by the histochemical and immunostimulatory together with plethysmographic studies.

**Conclusions:**

Thus, frog skin for the first time has been demonstrated to possess lipid components with pharmaceutical and therapeutic potential. The identification and characterization of such natural healing molecules and evaluating their mechanism of action would therefore provide basis for understanding the cues of Nature and hence can be used for application in medicine.

## Background

Wound healing is a complex phenomenon involving a highly dynamic integrated series of cellular, physiological and biochemical processes. In our search for wound healing mechanisms, a Naga based technique of using frog (*Hoplobatrachus sp*.) skin was found to be very effective. Acceleration of wound healing by the application of several biological membranes is very common owing to their efficacy in preventing infection and sepsis. However, frog skin plays a complex role in addition to helping in the haemostasis and mechanical protection to wound site as described by Purna Sai *et al.*, [1]. Hence the purification and characterization of the individual constituents of the frog skin and understanding the mechanism of biological activity has been attempted in a sequential manner. Four broad spectrum 11 and 12 residue novel antimicrobial peptides coined tigerinins have been already isolated by Purna Sai *et al*., [2] and Sitaram *et al*, [3] from the adrenalin stimulated skin secretions of the Indian frog *Rana tigerina*. The healing or repair process can be classified into three main overlapping and interrelating phases [4] viz., inflammatory phase involving alteration of capillary permeability, transudation and cellular migration followed by proliferative phase involving proliferation of fibroblasts, endothelial cells and epithelial cells in the injured area. Finally the remodelling phase where in the cell production is balanced by cell death, collagen production by degradation and resorption and capillary formation by capillary obliteration [5]. In combination with pain reduction and prevention of infection that form the principle features of healing wounds, frog skin lipid constituents play a significant role during the proliferative phase of wound healing [6]. The characterization of the lipid composition was also elucidated by Purna Sai and Mary Babu [6]. This paper deals with the composite role of frog skin lipids in wound healing and more specifically during inflammatory phase.

## Materials and methods

### Chemicals and Reagents

All chemicals and reagents other than the special chemicals mentioned were of analytical grade.

### Extraction of Skin Lipids

Skin peeled from the freshly chloroformed frogs was macerated and lyophilized to ensure the complete removal of moisture that contributes to 70% of the wet weight. The lipids were extracted from the lyophilized skin in chloroform: methanol (2:1 v/v) according to Folch *et al.*, [7] using Soxhlet apparatus. The extraction of the lipids was carried out at 50°C for 8 hours in a Soxhlet apparatus after which the thimble containing the skin pieces was removed and the extract was concentrated by evaporation of the solvent at 50°C. Final traces of the solvent were evaporated completely with nitrogen gas. The total extract obtained was then suspended in polysorbate 80 diluted 10 times with distilled water (1:10) that served as a vehicle for *in vivo *experiments.

### Composition of the lipid extract

The total extract obtained from the frog skin was analyzed to study the amounts of neutral lipids, phospholipids, free fatty acids, cholesterol, cholesterol esters, diglycerides and triglycerides present.

### Wound Healing Studies

Female albino rats of Wistar strain weighing 120 g were selected. Open excision type of wounds of a standard size (5 cm^2^) was created under mild anaesthesia as described in detail by Purna Sai *et al*., [1]. Three series of rats were maintained, one forming the control group and the other two series serving as experimental groups. One of the experimental groups was topically applied with the frog skin lipid extract, while the other group was injected intraperitoneally with varying concentrations (6, 12, 24 mg/Kg body weight) of lipid extract. The control group was similarly injected with polysorbate 80 that served as a vehicle. All experimental procedures were approved by the Animal Research Ethics Committee of Central leather research Institute (IAEC Registration No. 466/01/a/CPCSEA).

### Contraction Rate Measurement

The sizes of the wounds in both control and experimental animals were traced on a transparent graph paper and then analyzed in terms of area of the wound using a planimeter so as to compare the rate of contraction.

### Biochemical Characterization of the Wound Granulation Tissue

The wound granulation tissue was removed every alternate day from both the control and experimental animals. The hydroxyproline, hexosamine were estimated throughout the healing process in the granulation tissue as described by Woessner (1961) and Elson and Morgan(1993), respectively [8,9]. Uronic acid was also estimated as described by Schiller et al., and Bitter et al., [10,11]

## Anti-Inflammatory Studies

### Effect of Lipid Extract on Acute Inflammation

Female albino rats of the Wistar strain (120 g) were used in groups each comprising of 6 for the study. Pedal oedema was produced by injecting 0.05 ml of freshly prepared suspension of 1% carrageenan in the plantar surface of the right hind paw. Foot pad thickness was measured plethysmographically at regular intervals after the injection of carrageenan.

In one set of experiments, the lipid extract was administered intraperitoneally 1 hour before carrageenan injection as a suspension in polysorbate 80 which serves as a vehicle. In the second set of experiments, the lipid extract was injected 1 hour after the injection of carrageenan. Three doses of the lipid extract (6, 12 and 24 mg/Kg body weight) were selected for evaluating the anti-inflammatory effect and were compared with similar doses of known anti-inflammatory agent hydrocortisone.

### Histochemical Studies

At specific intervals (1 and 4 hrs) after the carrageenan induction of oedema, both the experimental and control rats were sacrificed and the plantar region of the paw was fixed in (10%) formalin, processed by routine histological procedures and subsequently embedded in paraffin. Serial sections were cut at 8-10 μ thickness and were stained with haematoxylin and eosin.

## Immunostimulatory Studies

### Preparation of the Antigen

The antigen was prepared according to Sharma *et al.*, [12]. Sheep erythrocytes were washed well three times in pyrogen free sterile normal saline and adjusted to a concentration of 5 × 10^9 ^SRBC/ml for immunization and challenge.

### Hypersensitivity Reaction (HR)

Hypersensitivity reaction to SRBC was induced in rats following the method of Doherty [13]. Groups of 6 rats each were immunized by injecting 0.1 ml of 5 × 10^9 ^SRBC/ml into the right hind foot pad on day 0 and challenged 7 days later by injecting intradermally the same amount of SRBC into the left hind foot pad. Thickness of the left hind foot pad was measured with a vernier callipers at 4 and 24 hours after challenge. Frog skin lipid extract was injected intraperitoneally in doses of 6 and 12 mg/Kg body weight on each of 2 days prior to immunization, on the day of immunization and on each of the 2 days after immunization (days -2, -1, 0, +1, +2) in the experimental group, while the control group was injected similarly with polysorbate 80.

### Humoral Antibody Response

Two groups of six rats each were immunized by injecting intraperitoneally 0.5 ml of 5 × 10^9 ^SRBC on day 0. Blood samples were collected from individual animals by the retro-orbital puncture on day 7 and day 14. Frog skin lipid extract was administered intraperitoneally on days (-2, -1, 0, +1, +2) in the group forming the experimental series, while the control group was similarly injected with polysorbate 80. Antibody response was observed by the haemagglutination technique.

## Results

### Composition of the lipid extract

The composition of the lipid extract as determined by us in our earlier paper [6] is indicated (Tables 1 and 2).

**Table 1:** Lipid composition of dorsal skin of *Hoplobatrachus *sp.^a^

Constituents	lipid extract (nmoles/ml)
Free Cholesterol	4,500
Cholesterol Ester	288
Diglycerides	1,905
Triglycerides	4,114
Fatty Acids	196
1)12:0^b^	16
2)14:00	12
3)16:00	112
4)18:0+18:1	50
5)18:02	6

^a^Amount of total lipids = 32.2 mg/ml

^b^Number of carbon atoms: Number of unsaturated bonds

**Table 2:** Phospholipids composition of dorsal skin of *Hoplobatrachus sp*.

Individual Phospholipids	Percentage of total phospholipids
Lysophosphatidyl choline	7.95
Phosphatidyl choline	31.45
Sphingomyelin	16.1
Phosphatidyl serine + Phosphatidyl Inositol	16.5
Phosphatidyl ethanolamine	28.1

Total Phospholipids: 4996 nmoles/ml lipid extract

### Effect of Lipid Extract on Wound Healing in Rats

Lipid extracts treated rats both topically and intraperitoneally healed completely by 12 days while the control rats took 20 days for healing. The wound healing observed was found to be dose dependent.

### Effect of Lipid Extract on Wound Contraction Rate

**Figure 1:**
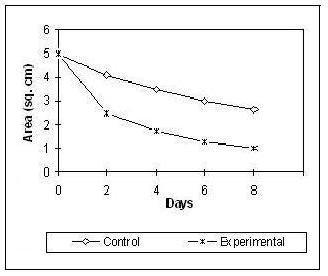
**Effect of topical application of lipid extract on contraction rate during the course of wound healing**.

**Figure 2:**
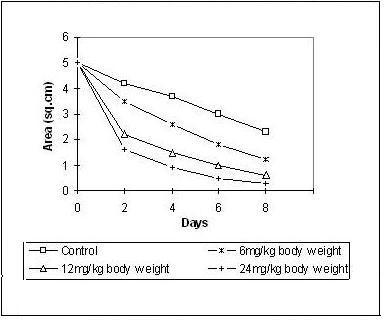
**Effect of intraperitoneal injection of lipid extract on contraction rate during the course of wound healing**.

The contraction measurements indicate a dose dependent reduction of the wound area. The rate of contraction in the experimental series was much faster and regular compared to the control group (Figures 1 and 2).

### Influence of Lipid Extract on Biochemical Characterization of the Granulation Tissue

**Figure 3:**
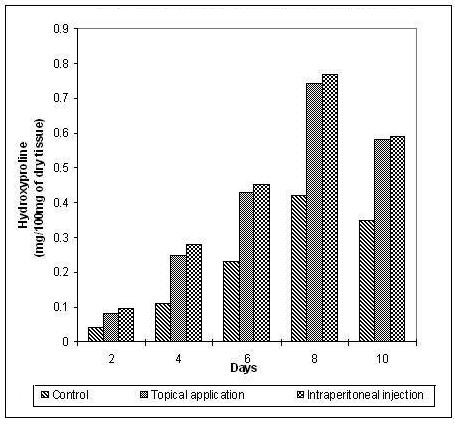
**Hydroxyproline content in the granulation tissue during the course of wound healing**.

**Figure 4:**
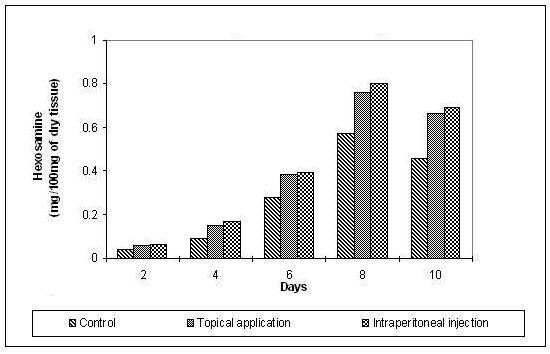
**Hexosamine content in the granulation tissue during the course of wound healing**.

**Figure 5:**
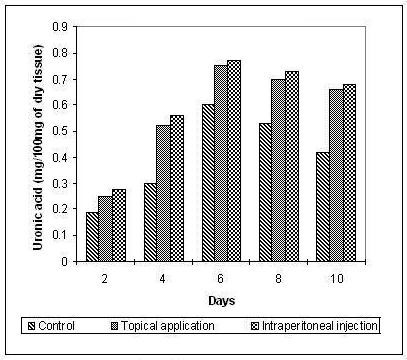
**Uronic acid content in the granulation tissue during the course of wound healing**.

The hydroxyproline and hexosamine content was found to increase gradually up to the 8th day and then decreased gradually. The increase in the hydroxyproline (Figure 3), hexosamine (Figure 4) and uronic acid (Figure 5) was more prominent and regular in the experimental series compared to the control.

### Effect of Lipid Extract on Acute Inflammation

A dose related decrease in the pedal oedema was observed as measured plethysmographically. The decrease in the oedema was significantly greater when the extract was injected prior to the injection of carrageenan. The reduction in pedal oedema was more in the lipid extract treated animals in comparison with the response to hydrocortisone which in turn was more efficient than the control (Tables 3 and 4).

**Table 3:** Effect of Lipid Extract administered intraperitoneally 1 hour before carrageenan induced rat pedal oedema at different time intervals.

Dose	2 HOURS	4 HOURS
Control - Polysorbate	0.77 ± 0.02	0.51 ± 0.04
Control + Polysorbate	0.76 ± 0.01	0.51 ± 0.01
		
**Lipid Extract**		
6 mg/kg	0.58 ± 0.03 *	0.30 ± 0.04 *
12 mg/kg	0.45 ± 0.01 *	0.18 ± 0.08 *
24 mg/kg	0.23 ± 0.03 *	0.10 ± 0.02 *
		
**Hydrocortisone**		
6 mg/kg	0.61 ± 0.02 *	0.35 ± 0.06 *
12 mg/kg	0.49 ± 0.03 *	0.23 ± 0.01 *
24 mg/kg	0.27 ± 0.04 *	0.15 ± 0.03 *

n = 6; * p < 0.05

Each value represents the increase in paw oedema in mm (Mean ± SE).

**Table 4:** Effect of Lipid Extract administered intraperitoneally 1 hour after carrageenan induced rat pedal oedema at different time intervals.

	2 HOURS	4 HOURS
Control - Polysorbate	0.77 ± 0.01	0.50 ± 0.08
Control +Polysorbate	0.77 ± 0.02	0.51 ± 0.01
		
**Lipid Extract**		
6 mg/kg	0.64 ± 0.01 *	0.39 ± 0.02 *
12 mg/kg	0.52 ± 0.04 *	0.24 ± 0.01 *
24 mg/kg	0.31 ± 0.04 *	0.11 ± 0.03 *
		
**Hydrocortisone**		
6 mg/kg	0.68 ± 0.02 *	0.40 ± 0.01 *
12 mg/kg	0.59 ± 0.03 *	0.34 ± 0.01 *
24 mg/kg	0.40 ± 0.06 *	0.22 ± 0.03 *

n = 6; * p < 0.05

Each value represents the increase in paw oedema in mm (Mean ± SE).

### Histochemical Studies

**Figure 6:**
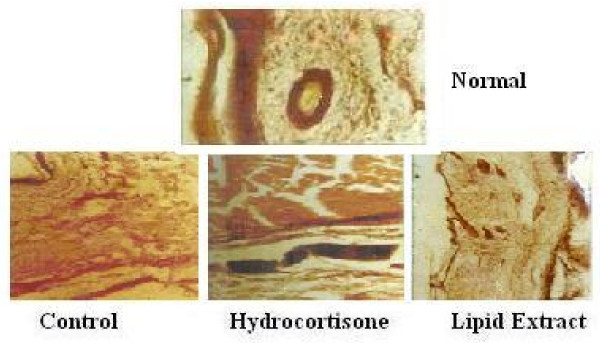
**Onset of the inflammatory cell infiltration in the control, hydrocortisone, and lipid extract treated rat paws respectively one hour after the carrageenan injection**.

**Figure 7:**
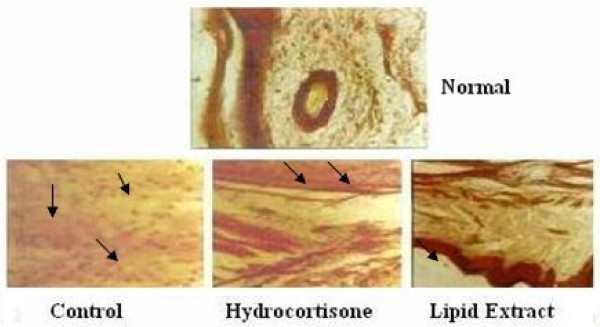
**Comparison of the decrease in oedema in the control, hydrocortisone and lipid extract treated animals four hours after carrageenan induced oedema**.

Histological and histochemical studies of the rat paws showed stratified squamous epithelium with the underlying sub-epidermal region consisting of capillaries and muscles. The onset of inflammation upon the injection of carrageenan is indicated in the control, hydrocortisone, lipid extract treated animals (Figure 6). An intense infiltration of inflammatory cells (mono-nucleocytes and lymphocytes) in the control extending up to the bony trabeculae was observed, while the hydrocortisone and lipid treated sections indicated a moderate infiltration of inflammatory cells (Figure 7). However, the infiltration of inflammatory cells extended up to the bone in the hydrocortisone treated and the control rats whereas in the lipid extract treated rats the impact was restricted to the muscle level. A gradual decrease in the pedal oedema was observed by the 4th hour in the control, hydrocortisone treated and lipid treated rats respectively. The rate of decrease in the oedema as observed by the distribution of the inflammatory cells was greater in the extract treated rats than the hydrocortisone treated rats which in turn were greater than the control.

### Hypersensitivity Reactions

Intraperitoneal administration of lipid extract (6,12 mg/Kg body weight) for 5 days around immunization produced a dose related increase in early (4 hrs) and delayed (24 hrs) hypersensitivity reactions in rats (Table 5).

**Table 5:** Effect of Lipid extract on early (4 hr) and Delayed (24 hr) Hypersensitivity Reaction.

Inflammation after challenge - Foot pad thickness (mm)		
**Dose (mg/kg)**	**4 hr**	**24 hr**
Control	3.16 ± 0.02	3.09 ± 0.04
		
**Lipid Extract**		
6	3.38 ± 0.06	2.90 ± 0.04 *
12	3.65 ± 0.03	2.75 ± 0.02 *

Values are Mean + SE of six samples

* p < 0.05.

### Humoral Antibody Response

**Figure 8:**
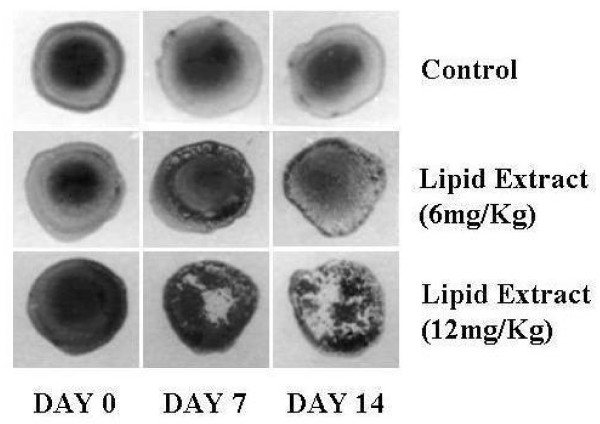
**Effect of the lipid extract on inflammation observed through haemagglutination**.

A dose related increase in humoral antibody response (Figure 8) was observed from the haemagglutination in the rats treated with the lipid extract (6, 12 mg/Kg) for 5 days around immunization. Further, agglutination reaction was more intense on the 14th day when compared to the 7th day.

## Discussion

The application of animal fat, ox grease, lion grease etc., for wound healing was prevalent since several centuries in Egyptian medicine [14]. The results of the above study indicated that one such animal fat viz., lipids of frog skin, whether applied topically or injected intraperitoneally accelerate the healing of wounds suggesting a scientific basis for the traditional medicines.

The significant increase in the hydroxyproline, hexosamine and uronic acid in the early days of healing followed by a decrease in the later days ensures the maintenance of equilibrium between synthesis and degradation of the extracellular matrix proteins so that the remodelling of the wounds is achieved properly thereby reducing scar formation.

Oedema induced by carrageenan is a model of exudate inflammation and agents effective in decreasing carrageenan induced oedema can be used as anti-inflammatory agents in acute inflammation. Carrageenan induced oedema is mediated through histamine and serotonin in the first hour followed by kinin release up to two and half hours and lastly by prostaglandins from two and half to six hours [15,16]. The lipid extract indicated a dose dependent activity against the three mediator phases as observed by the reduction in the pedal oedema. The lipid extract has been completely characterized by Purna Sai et al., (1998) [6] majority of which is composed of phospholipids. Further, the occurrence of antihistaminic principles in the *Hoplobatrachus sp*. tissues, more specifically skin as reported by Jayasunder *et al*., (1973) [17] support the present findings. The fact that the lipid extract was able to reduce the inflammation even when injected after the onset of the inflammatory process suggests that it serves as a good anti-inflammatory agent.

Immunostimulatory studies indicated that lipid extract stimulates both the humoral and cell mediated responses in the experimental animals. Increase in the DTH reaction in rats in response to thymus dependent antigen revealed the stimulating effect of lipid extract on lymphocytes and accessory cell types required for the expression of the reaction.

The augmentation of the humoral response to SRBC by the lipid extract as evidenced by the increased haemagglutination indicated the enhanced responsiveness of macrophages and lymphocytes involved in the antibody synthesis. In view of the key role played by macrophages in co-ordinating the processing and presentation of antigen to lymphocytes, the augmentation of the humoral response to SRBC reveals that lipid extract may enhance the effect by facilitating these processes.

These studies suggest that any agent capable of accelerating any of the phases of wound healing (inflammatory, proliferative and re-epithelialization phases) would result in more rapid healing thereby reducing scar formation.

## Conclusions

In conclusion, the frog skin lipid extract irrespective of the mode, whether topically applied or injected intraperitoneally accelerate healing. The influence of the lipid extract on acute inflammation and immunostimulatory response indicated that the mechanism of quickened healing could be attributed partly to the reduction in the inflammatory phase that corresponds to the prime phase in wound healing. Thus, the frog skin lipid extract enhances the healing by playing a pivotal role in the first two phases of healing although it did not possess any significant antimicrobial effect. These studies therefore confirm the scientific significance of frog skin and its constituents in wound healing. This may be attributed to the fact that the amphibians being the first true land vertebrates connecting land and water obviously developed certain evolutionary specializations for regeneration and repair so as to withstand the onslaught of predators causing frequent injuries if not killing them *in toto*. Hence, exploring the mechanisms of the bioactive constituents would enable the use of these cues to achieve multilayered cell engineering regimens to produce more complex tissue architectures. This would lead to an increase in sophisticated biotechnological strategies for the improvement of techniques to engineer bio-artificial grafts and implants for burns and wound injuries in particular.

## Abbreviations

DTH: Delayed Type Hypersensitivity; SRBC: Sheep Red Blood Cells.

## Competing interests

The authors declare that they have no competing interests.

## Authors' contributions

VK carried out the experiments and involved in drafting the manuscript. MB was involved in reviewing the manuscript. RR is the head of the department involved in general scientific discussions and PK conceived, designed the study and was instrumental in coordinating as well as drafting the manuscript. All the authors read and approved the final manuscript.
